# Critical Remarks on the Disease of Count Cavour, and Its Treatment

**Published:** 1862-01

**Authors:** Stephen Rogers

**Affiliations:** Late Surgeon to the Panama Railroad Company; Licentiate of the Royal University of the Havana (Cuba) Medical Department, etc., etc.


### CRITICAL REMARKS ON THE DISEASE OF COUNT CAVOUR, AND ITS TREATMENT.

BY STEPHEN ROGERS, M. D.,

*Late Surgeon to the Panama Railroad Company; Licentiate of the Royal University of the Havana (Cuba) Medical Department, etc., etc.*

The subject of the untimely demise of the great European statesman, Count Cavour, and of the professional discussions *pro* and *con* upon the medical management of his fatal disease, have been worn well-nigh threadbare by both the medical journals and the newspapers all over the world.

Europeans, perhaps, have come to some conclusion satisfactory to themselves in the affair; and the profession on this side of the ocean, very likely, have, as a general thing, concluded that at this distance and with the indefinite accounts received, we have little data for a well-founded criticism upon the professional behavior of the Count's physicians during, and of their scientific treatment of, his last illness. I confess to have been one of this generality up to the time of the appearance in your journal, of the translated clinical report of Count Cavour's last sickness and death, from *L'Union Medicale*, by Dr. Deslandes. To the American physician, particularly if he has seen much of miasmatic disease in the tropical regions of this continent, or in those sections bordering upon the tropics, and to the young men who are just starting in their career, and who may be thrown into these regions, this clinical, and as stated *reliable* report, must, it appears to me, prove a very curious, interesting, and—as I hope to make it—a very instructive and useful one. Let us commence, then, with the first important point touched upon by the report, viz: the habits of life and occupation of the patient. His mental labor, it is stated, had been for many years excessive, particularly during the last two years, a manner of life well known to be inconsistent with a high grade of animal vitality, and a fact which should have had more respect shown it by the physicians who treated his disease. With this preparatory information, it is no matter of surprise that gout should have troubled him occasionally, nor that he should so long ago as years prior to his death, have had so much trouble to get rid of an intermittent fever as the report states he had. The fact of his not having suffered any "serious or long disease,"

cannot necessarily interfere with these conclusions, for one's *vis medicatrix naturæ* is not by any means shown by a long exemption from disease, irrespective of circumstances.

Expose an individual to causes of disease, to which every body about him, as a rule, yields, then if he escape, it is very safe to accredit him with an extraordinary amount of vital protective force, or in common language, with a fine constitution. Count Cavour, so far as we know, was never thus accredited. On the contrary, we have the statement, that he threw off disease badly, in the history of his long battle with the intermittent fever of six years before. This is the most reliable means of estimating vital force and strength of constitution, viz: the promptness with which disease is thrown off, and with which the system recovers from its effects. With the light of these truths before him, any observing physician could not but have taken the patient in question, as a case requiring cautious and perhaps sustaining treatment, rather than the routine medication and haphazard depletion which we shall see he was subjected to. We now come to the—strictly speaking—commencement of the disease, which finally terminated so unfortunately, viz: the frequent attacks of colic, which are said to have troubled him for about a year prior to his final colic, which we shall hear more of presently. Although purporting to be a clinical record of what actually took place, a more indefinite, objectless, and unsatisfactory collection of words, cannot be conceived of than this paragraph. Absolutely nothing of import is communicated, except perhaps that he had little confidence in his physicians, which we may yet see reason for thinking was not at all strange. We are told that "for about one year he had been complaining of very sharp colics, coming on at night usually, and which *he* treated by *one* or *two* bleedings." Not a word is said of the accompanying symptoms; if there were headache, febrile state of the skin, peculiarity of the pulse, coolness or blueness of the surface; or if these attacks passed off without either heat or perspiration, or if they observed any kind of periodicity in their nocturnal attacks. And if so, whether they were quotidian, tertian, or quartan attacks; nor of the intervals between the attacks. But fortunately, a little further on, we find what may help us to a solution of the doubtful points. "On the 29th of May," says the report, "in the evening, he was *again* seized with colic." This expression leads us to the conclusion, that this seizure was not unlike, if it was not precisely like the previous ones, which we have just seen he had suffered for a year, more or less. Here also we are left in complete ignorance as to any attending phenomena of the colic, if there was any headache, any peculiarity in the condition of the skin, pulse, or temperature. Almost as if by eccident, we are finally told, that some time the following day "*the fever being intense, it was thought necessary to bleed him again* TWICE."

After the bleeding, which had now been practiced three times since his attack of the previous evening—and which really seems to be the object of the report to record—we know nothing of his symptoms, till the morning of the 31st, at least thirty hours after his seizure by the colic. It may have been thirty-six hours. He was then in the language of the report, with the "apyrexia complete." Here also occurs a statement, which fixes in my mind a deep conviction that the Count's physician, however meritorious a man, was utterly worthless to him as a medical adviser. It is this: "M. de Cavour, thinking himself cured, acted accordingly." I also take it as giving another key to the hidden history of his previous colics. It seems to say that he had suffered similar seizures frequently, and they had terminated as this had, and as the probabilities of a recurrence—judging from his previous experience—were considered slight, he discharged his physician, or what amounts to the same thing, refused to be governed by his advice. I submit the opinion that there is the best reason for believing that his previous colics had been more or less like this one, during the whole year before, and that the case up to the 31st of May, had been nothing else than an irregular and more or less marked intermittent fever. From the fact that after all this fever of the night of the 30th of May, no medicine was prescribed that we have any account of, nor any recommended, I am forced to the conclusion, that up to this date the Count's physician did not comprehend his patient's disease, or if he had an inkling of its true character he was overruled by, and very culpably submitted to the opinions and will of his illustrious patron. We are not told if his physician had even seen him in any of his previous attacks of colic, which one or two bleedings are said to have always cured. However this may have been, it is scarcely credible that he could have attended a patient so intelligent as this one, through such an attack as was experienced on the 29th and 30th, without making the discovery that he had frequently suffered similar ones. But if with all this history, and this last day's experience, the case was not understood by him, and he was unable to make anything out of it, his responsibility is somewhat lightened, for after all it is hard to blame one for the mismanagement of a thing he does not understand. But if he did comprehend the facts in the case, and still gave his assent to what he must have known were erroneous and dangerous notions of his patient, he is in a high degree censurable. His course was plainly to have resigned his charge, as soon as his patient refused to be governed by his important instructions. As to the real character of the colic, so much spoken of, the probabilities are very strong, judging from all the circumstantial evidence we are able to collect, that it was simply *gastric* pain, not by any means an unheard of attendant on, and sometimes takes the place of the cold stage of intermittent fever. Whatever may have been the facts previously, we now see that it is no longer possible to mistake the character of the Count's disease, for on the evening of the same day, the 31st, in the morning of which he considered himself cured, a period of forty-eight hours from his last seizure by colic, we are informed that "*a new attack came on with reaction towards the brain.*"

All that we have thus far gone over, is equalled in indefiniteness, unscientific looseness, and downright stupidity, only by this statement. What do we learn by it? That he was attacked again by colic? That he was taken with chill? That he was seized with delirium? Or that he had any of the early or later symptoms of intermittent fever? To all this, the report only replies, "the abdomen was painless on pressure," and that at his *own* request the patient was bled. Whether the reaction spoken of means to say he suffered headache, or was delirious, it is impossible to say; but if the former, it was precisely what might have been expected after so many bleedings; if the latter, he certainly was in a very unfit condition to give so important directions, no matter what his medical education may have been: so that in either case, his physician was again guilty of a dangerous inefficiency, in not having been governed by his own judgment, which, thus far, we have not seen that he was, in a single instance. At this stage of the drama he was bled twice again, and then nothing more is said of him for thirty-six hours more or less, when we are told that during all that period he had not slept. Had he been delirious during a great part of this time? How many hours of it had his fever been intense? What remedial measures had been employed besides the bleeding? No reply to any of these inquiries; but the probabilities are strong certainly, that, what with his exhausting bleedings, and his depressing miasmatic disease, he must have been, a part of the time at least, in a condition bordering upon asthenic delirium, if he was not absolutely crazy. The next therapeutical proceeding recorded is "an injunction;" but for what purpose it was given or of what composed, we know as little as if it were not mentioned at all.

It now for the first time appears that the physician suspected there might occur another paroxysm, or, as it is termed in the report, "an exacerbation," a singular name, by the way, for a paroxysm, for we are told in the context, as plainly as it would seem possible for this reporter to tell anything, that another paroxysm did come on the following evening, and that the patient was free from fever through the day.

Here for the first time, after two severe and long paroxysms, we learn that treatment was commenced. In reply to the query put in the report, "Could this treatment have been commenced too late?" I would say, that *treatment* should have been commenced much earlier; but even at the time mentioned, I do not think the case was by any means desperate, had it been attacked in an efficient manner, which was by no means the case. The doses were entirely inadequate, and given in a most routine empirical manner. I cannot better illustrate the different results obtained from quinine, administered in the doses given to Count Cavour, and those obtained from such doses as experience the world over has shown proper, than by quoting the written report upon this point, of one of the surgeons of the U. S. Army. He says, "some two years since I was so unsuccessful in arresting the paroxysms of intermittent with the sulphate of quinine, given in two-grain doses every hour, (although during the apyrexia as much as twelve, eighteen, or twenty-four grains had been given,) that I laid it by in despair, and resorted to sedatives and relaxants, such as tartrate of antimony, ipecac, opium, etc. Still, however, I was not satisfied, and the great reputation the Peruvian bark had so long enjoyed, created doubts as to the propriety of abandoning its use. Soon, therefore, I determined to give it another trial in larger doses, and with this view I commenced three or four hours before the expected paroxysms, and gave from four to six grains every hour until it produced its peculiar effects upon the brain—ringing and buzzing sounds in the ears, a sense of stricture across the forehead, and temporary deafness—effects invariably produced in every case where three or four such doses had been given. From this time forward I was constantly successful, nor do I remember a case in which it failed, when the peculiar effects it displays on the nervous system were produced. Finding, then, that the enlarged doses had such happy effects, I was induced in many cases, when the apyrexia was short, to give it in single doses of from ten to fifteen or twenty grains, according to the violence of the disease. Here, then, I saw cases of intermittent fever that could not be arrested by fifteen or twenty grains of sulphate of quinine, given in small and divided doses, yield immediately to the same quantity given in larger doses at much shorter intervals."*

As to the comparative activity of the citrate and sulphate of quinine, I have never taken the pains to determine by experiment, but can see no reason why there should be any perceptible difference. But whether there be or not, is unimportant in this particular case, for Cavour did not take enough, had it been the *sulphate*, to produce any results. The gentleman above quoted utterly failed to break up paroxysms with it, given at the rate of two grains every hour; but the Count took of the first prescription only two and a half grains every two hours, really about half the amount. Later we are told that it was prescribed in five-grain doses, but as it was so timed that he could take only one dose before the beginning of another paroxysm, it turned out to be useless, and the whole fifteen grains were thrown away. There is another singular circumstance brought out in this record, viz: that the patient never had *chills*, till some hours after he had commenced to take the quinine. This, for all the world, looks like a case made up to suit the medication.

However, he had a chill, at last, of an hour's duration, followed by fever which lasted for twenty-four hours, more or less intense, attended with delirium. Here we have a positive declaration, that in *a state of delirium* the patient insisted on being bled, and accordingly *was* bled for the sixth time. If this is a veritable statement of what occurred in the sick-room of this patient, more convincing evidence of the imbecility of Count Cavour's physician can hardly be conceived.

Should I go on in this order of examination, little else than a repetition of the same puerile proceedings could be found throughout this most extraordinary clinic. We will not, therefore, spend more time with what was done or said to have been done, but proceed to consider what might and should

* Medical Statistics U. S. Army, 1839 to 1854, page 638.

have been done. Well may the author of this clinical report ask, "Now what was the disease?" If the Count's physician made up this report, he can hardly be charged with want of ingenuity, for a more ingnious mystification, and evasion of the main points in the case, would be difficult to match in the annals of either medicine or law, both so famous for this species of dodging. But with all the apparent effort to make a report, and say nothing, enough has forced itself into view to enable one who has seen much of miasmatic disease, to satisfy himself that the Count's disease was an *intermittent fever*. First, six years prior to this last illness, he had suffered much from, and was a long time getting rid of, an intermittent fever. This fact alone should facilitate greatly the diagnosis of any subsequent disease, having anything like a periodicity, as was doubtless the case with the colics of this patient, which we are told were particularly toublesome for the last year of his life. I have already given my reasons for believing that these colics had invariably been attended with fever. Second, about two weeks prior to his final illness, he had exposed himself for some days to great heat of sun in riding over his country estates, and—although nothing is said of it—probably to miasmatic exhalations. For a week or ten days after his return from this fresh exposure "he was observed," says the report, "not to be so well as usual, and more irritable." This moral condition is a most common premonition of miasmatic fever, as well as an accompaniment of the incubation (if this term may be employed) and development of miasmatic disease. Third, on the night of the 29th of May, he was seized with one of his *accustomed* colics, which terminated in fever followed by distinct apyrexia, as we have seen. Forty-eight hours after, another paroxysm set in, ran its course, and was followed at the same interval by a third, setting all doubts as to its character at rest, even in the minds of his tardy physicians, who it appears did not commence their trifling medication till he had suffered two severe paroxysms, and had been bled five times. Had the patient, on the morning of the 29th, when seized with colic as it is called, been ordered into hot water to the knees, and opium sufficient to relieve his gastric pain, with acetate of potash, or spirits of nitre, or small and frequent doses of ipecac, to induce and promote perspiration, the hot stage of the paroxysm would, without the least doubt in my mind, have been curtailed to less than six hours, while the various bleedings so reduced the vital forces, that his brain was desperately at work for twenty-four hours, battling with the disease, and restoring the system to a normal state.

As to the amount of blood taken, we are quite as ignorant as on any other important point in this most dubious report. But whether much or little, I do not hesitate in declaring, that every drop taken, did the patient injury. Plethoric butchers may occasionally be bled in miasmatic fever without danger, but a man of the age, the habits, and the brains of Cavour, loses blood badly in these diseases, at great expense of vitality, and much at his peril. At the commencement of the apylexia on the morning of the 31st, or even after the second paroxysm, and all of the bleedings, on the morning of the 2d of June, as soon as the skin began to feel moist, which, by the way, I believe is not mentioned in the whole report—ten grains of quinine in solution should have been administered, and repeated hourly till his ears rang, or till he showed some other evidence of being thoroughly under its influence, and then should have been kept so far at least forty-eight hours, by a dose once in five or six hours of sufficient amount.

Here I wish to remark, that my very extensive experience in the use of quinine has convinced me that the theoretical administration of it, in small and repeated doses, for the object of saturating the system, and thereby expelling, or neutralizing, or destroying the miasm, is an unreliable, generally useless, and dangerous practice, in districts where there exists much intensity of miasmatic poison. A dose of quinine so small that it produces no sensible influence or impresaion, is not to be depended upon either as a preventive or curative. The quantity requisite to produce this effect, is variable, from ten grains down to three. As a general rule for adults, four or five grains will produce it (if given in solution), but if not, it should be repeated in the course of an hour. It seems hardly requisite to say, that when a prompt action of this agent is required, it should be given in solution. Quinine is not a cumulative medicine. Its effects are about as transitory, and it is eliminated from the system with as great facility and rapidity, as a dose of alcohol. Hence, if the indications of its influence upon the brain—such as buzzing in the ears, etc., are established, and allowed to pass off, before the hour for the commencement of a new paroxysm, it will rarely do any good. I have repeatedly, in my own person, failed to arrest premonitory symptoms of miasmatic fever—such as pains of the extremities, loss of appetite, restless nights, slow digestion, etc., for two or three days in succession, by the occasional use of a moderate dose—say four or five grains—which at once disappeared, the moment my ears began to ring from a double dose. I have never seen any reason to believe in the chemico-therapeutic doctrine, that the presence of quinine in any quantity, and miasmatic poison in a state of activity, cannot at the same time exist in the system; that the quinine neutralizes the poison, in some inexplicable chemico-vital manner. It exerts its medicinial power, by simply stimulating the brain to a more or less healthy vital action, of resistance, restoration, and preservation. Hence if it be not administered in amount sufficient to produce effects appreciable by the senses, it will, as a rule, be worthless in the treatment of intermittents. From this belief, which I have not settled down in without ample evidence, comes my opinion, that if Cavour, on the morning of the 31st May, had taken ten grains of either citrate or sulphate of quinine in solution, and if his ears did not, in the course of an hour, begin to give indication of its action, repeated the dose, and so on, till he *had* unquestionable evidence of its action, by occasional doses, for two days; notwithstanding the three suicidal bleedings he had already suffered; his disease would have been cured, or at least that he would have recovered from that attack, and his country be spared her loss. At the approach of the hour for the paroxysm, which in order would have come on the 4th June, it would have been prudeut to take another ten grains for safety. It is always safe practice in miasmatic disease, to particularly respect the first and second regular periods, after the one he has been carried over by antiperiodic, and to show this respect, by administering a good dose, of from five to ten grains, two hours in anticipation of its arrival.

Intermittent clearly, and simply in the beginning, Cavour's disease, by repeated bleedings, and by the exhaustion of vital force, produced by the successive uninterrupted paroxysms of a violent fever, was converted into what the reporter calls *ataxic*, attended very naturally by the delirium of exhaustion. Hence we see, that during the remainder of his existence, after the sixth bleeding, he was more or less constantly delirious. It was in almost every particular a case of what in this country is known, or supposed to be known, by the name of Panama or Chagres fever. The main point of difference is, that in his case vitality was exhausted by *bleeding* and fever, while in the latter it is reduced by the influence of tropical *heat*, and fever. The suggestions as to the treatment that should have been employed, both in the paroxysms and to prevent them, in this case, are an expression of the conclusions arrived at, from an extensive experience with this last named fever.

If these remarks and opinions, frankly given, result in throwing any light on this—as already stated—nearly worn out subject; and especially if they are ever of any service to the young physicians, who are annually going forth from this country to all parts of the world; my wishes are attained. I desire to impress them with the fact, that next to ignorance, comes inefficiency in administration, and that if they get patients whom they cannot control, it is much better for their professional reputation, and peace of mind, to resign the care of them, than to be dragged by them into discredit, and perhaps infamy, for an act which may be the fruit of ignorance or indiscretion, on the part of the patient, if assented to by the physician, who understands the dangers attending it may render him highly censurable, criminal, or even infamous.

Count Cavour is past recovery; let this example serve as a lesson for future reference, to all whom it may interest.

New York, Nov. 18th, 1861, 42 W. 29th St.—*Am. Med. Times.*

